# Optimizing long-acting injectable antipsychotic safety and care continuity through documentation best practices

**DOI:** 10.3389/fpsyt.2025.1659290

**Published:** 2025-09-01

**Authors:** Amie R. Throneberry, Bradley G. Burk, Brandon S. Pruett

**Affiliations:** ^1^ Department of Clinical Pharmacy & Translational Science, University of Tennessee Health Science Center College of Pharmacy, Nashville, TN, United States; ^2^ Department of Pharmacy, University of Alabama at Birmingham Medical Center, Birmingham, AL, United States; ^3^ Department of Psychiatry and Behavioral Neurobiology, University of Alabama Max Heersink School of Medicine, Birmingham, AL, United States

**Keywords:** documentation, long-acting injectable antipsychotics, medication reconciliation, antipsychotic depots, EHR interoperability, best practices, transitions of care, psychiatric medication safety

## Abstract

**Background:**

Long-acting injectable antipsychotics (LAI-APs) are vital for managing psychiatric conditions, particularly in patients with adherence challenges. However, errors in medication reconciliation, prescribing, preparation, and administration, often stemming from documentation lapses and communication breakdowns, may result in an increased risk of relapse or readmission. Despite their widespread use, standardized documentation practices remain underdeveloped.

**Objective:**

To identify documentation insufficiencies related to LAI-AP use across healthcare settings and propose best practice recommendations to improve safety and care continuity.

**Methods:**

A comprehensive review of literature using PubMed, Google Scholar, current clinical guidelines, and gray literature was conducted to identify safety concerns and documentation gaps related to LAI-APs. Search terms included “LAI antipsychotic medication errors,” “documentation,” “safety,” “mental health,” and “medication reconciliation.” Further articles were identified through a scan of the references cited within the initial sources. From this analysis, a series of best practice recommendations were developed targeting key aspects of LAI-AP use, with a focus on practical implementation strategies.

**Results:**

Limited literature is available on errors related to LAI-AP with minimal detail on documentation strategies to address these. By reviewing reports, extrapolating recommendations from their oral antipsychotic counterparts, and analyzing potential risk factors unique to the behavioral health setting, key documentation gaps were identified across the LAI-AP use process. These include deficiencies in medication reconciliation—especially related to last doses and oral overlap—along with inconsistencies in prescribing practices, patient education, preparation, administration, and transitions of care. Inadequate documentation contributes to missed or duplicate doses, incorrect administration techniques, and poor coordination between settings. Our findings indicate the need for standardized order sets, readily accessible documentation of initiation or discontinuation reasoning, integration of therapeutic drug monitoring and symptom tracking tools, structured patient education, and formalized transitions of care procedures.

**Conclusions:**

Enhancing documentation throughout the LAI-AP care continuum is crucial to reduce medication errors and improve patient outcomes. The proposed framework offers practical steps for clinicians and health systems to standardize documentation, improve communication during care transitions, and promote safer LAI-AP use. Innovations such as national electronic health records, EHR-integrated LAI-AP registries, and smartphone apps could facilitate cross-setting communication, patient engagement, and error reduction. Addressing systemic barriers will require policy-level reform.

## Introduction

Given recent publications emphasizing the utility of LAI-APs even for patients with first-episode psychosis (FEP), clinicians may see an uptick in usage of these medications (1–3). Further, off-label use of LAI-APs and FDA approval of various LAI-APs for bipolar disorder or more complex patient cases, including those with multiple comorbidities or dual LAI-AP use, has been increasing, illustrating the necessity for appropriate chronicling (4, 5). Presently however, guideline mentioning of LAI-AP is limited, and although there exists broad use of these high-risk formulations, there has been sparse guidance related to documentation or effort to reduce medication errors (6). One potential reason this guidance has not been prioritized may be related to the overall lack of literature surrounding LAI-AP errors, which is likely due to a deficit in voluntary reporting and not secondary to an infrequency of errors occurring (7, 8). Inpatient behavioral health units have been found to have poorly developed processes for effective communication and safe medication management support (9). The majority of errors in community and hospital behavioral health settings were related to aspects of prescribing or administration, including incorrect time, incorrect dose, or missed doses.

In 2023, the Institute for Safe Medication Practices (ISMP) released a newsletter highlighting reported safety issues with long-acting injectable antipsychotics (LAI-APs) (10). Among the safety concerns were complications related to LAI-AP medication reconciliation, formulation, multiple-dose vials, duplication, dose preparation, and administration technique. A substantial portion of these errors were related to a lack of documentation and/or communication. To date, a total of 16 different LAI-APs are FDA approved. While LAI-APs undoubtedly play an integral role in treating patients with mental illness (especially for those who are non-adherent to oral medication), the ramifications of erroneous duplications in administration can be significant. Following administration, LAI-APs provide a steady concentration of medication over a prolonged period. The duration for which these injectables cover has extended greatly, to a more recent six-month coverage period with Invega Hafyera. Although the result of accidentally giving two six-month shots in proximity may be akin to the accidental administration of two doses of oral antipsychotic administered together, the potential side effects incurred with the LAI-AP may be prolonged and intractable. Some instances of patients unintentionally receiving duplicate LAI-APs due to interoperability issues have been described (7, 10).

A literature review examining medication-related issues in the behavioral health setting found procedural errors such as a lack of administration route documentation contributed to the overall prescribing errors on behavioral health units (7). Documentation errors for prescribing were more common than clinical prescribing errors with a rate of 4–5 per patient. In a separate study analyzing the prevalence and predictors of prescribing errors across three behavioral health facilities, the authors found an error rate of 6.3%, with 56% deemed clinically relevant and almost 7% potentially serious or life-threatening (11). Most were associated with drug omission and incomplete or incorrect prescription requirements. These included an intramuscular LAI-AP being prescribed as subcutaneous, among other LAI-AP-related oversight. Associations have been discovered specifically between LAI-APs and drug omission along with a three-fold increased risk for wrong dose-related errors (12). Research on the standardization and implementation of prescribing, administering, and monitoring medicines is highly needed to successfully reduce medication errors within behavioral health settings (7).

Recently, there has been growing emphasis on the critical role of discharge planning, transitions of care, and comprehensive documentation in supporting continuity of care and adherence to LAI-APs (13). With varying electronic health records (EHRs) and healthcare settings in which LAI-APs are delivered, establishing best practices for LAI-AP documentation would allow for some standardization across care transitions and potentially put patients at a lower risk of experiencing a medication-related error. In this article we outlay many of the potential variables wherein improper LAI-AP documentation may occur leading to fallible administrations. We attempt to provide *Best Practice* recommendations for clinician adherence to maintain the highest patient care standards and avoid medication discrepancies. While some *Best Practices* may be derivative of those from oral medications, highlighting nuances for LAI-APs can help practitioners realize the specific intricacies.

## Medication reconciliation at admission

### Reasoning

Medication reconciliation represents a crucial step of the patient care process during hospital admission. Oversight or an incomplete medication reconciliation during the patient’s initial presentation can lead to a cascade of mismanagements throughout their treatment (14). In efforts to expedite this process while preserving comprehensiveness, the standardization of medication reconciliation documentation has been supported by federal and world agencies (15, 16). Still, despite this and other Joint Commission recommendations, around 40% of medication errors happen during the transitions of care process due to inadequate or unavailable medication reconciliations (17, 18). Even during internal hospital transfers there exists a reported 62% incidence of medication discrepancy errors (19).

This issue has been shown to have a high prevalence in the behavioral health setting, with medication errors identified in as many as 89% of adult patients boarding in the ED with a psychiatric-related chief complaint (20). Medications reviewed upon admission were five times more likely to be related to significant clinical errors compared to those checked during their stay (7). Although evidence linking effective medication reconciliation to outcomes beyond reducing medication errors is unclear, one systematic review found that pharmacist-led reconciliation programs were associated with significantly lower risks of adverse drug event-related hospital visits (RR 0.33; 95% CI 0.20–0.53) and hospital readmissions (RR 0.81; 95% CI 0.70–0.95) compared to usual care (21, 22). Interventions included comprehensive medication reconciliation during care transitions, follow-up phone calls, and/or patient counseling.

Lack of time was identified as the largest barrier to completing a comprehensive medication reconciliation, with less than 25% of emergency department (ED) nurses and physicians surveyed spending more than 10 minutes to conduct the reconciliation (23). Pharmacists have also reported time constraints as a barrier to medication reconciliation completion (24, 25). The time necessary to interview patients in the behavioral health setting can be further prolonged due to the challenging nature of patients with severe mental health diagnoses involving acute psychosis, advanced neurocognitive disorders, lack of insight, or lower health literacy (26, 27). Many times, secondary sources may need to be utilized to gain collateral information or confirm a patient’s history including recent admissions, prescription fills, and administration of medications (14). Examples of secondary sources may include family/caregivers, primary care providers, and outpatient pharmacies. In some instances, external medication histories are connected to the electronic health record (28). These data draw from all prescribers and participating pharmacies where the patient has filled their medication. Here, the LAI-AP may appear to have been filled, but the data do not show whether the patient has picked up the medication or if it has been administered, giving clinicians a false sense of security. Further, medications dispensed as part of an LAI-AP free-drug program or patient assistance program and those not claimed on insurance, through a Pharmacy Benefit Manager, or paid for using cash, may not be captured.

While a thorough medication history should be obtained on all patients, special focus should be placed on high-risk patients such as those with low health literacy, an inability to perform ADLs, a lack of psychosocial or family support, multiple comorbidities, cognitive impairment, recent psychiatric admissions, a history of medication non-adherence, or extensive medication lists (29). ISMP Canada recommends creating a random sampling process for selecting 20 patients per month to audit and review medication reconciliations (30). After auditing, a root cause analysis can be performed on any absent or incomplete medication reconciliation documentation. Trending risk factors for an insufficient medication reconciliation may aid in both patient characteristic and process-specific targets for concentrating efforts.

As stated later within the *Transitions of Care* section, research regarding the revolving door (RD) phenomenon risk factors have largely concentrated on patient characteristics. Since patients experiencing RD patterns transition more frequently between various inpatient clinical settings and other facilities that may lack interoperability, information regarding last dose administration of an LAI-AP may not be easily attainable. Therefore, RD pattern-associated risk factors may also serve as target populations for integrating focused communication and documentation efforts when only limited time is available. Putting forth the effort to gain this collateral information early on in a patient’s admittance to mental health services and documenting these findings appropriately may prevent duplication of these processes and reduce the risk of medication errors occurring.

### Best practice

**Figure 1:**
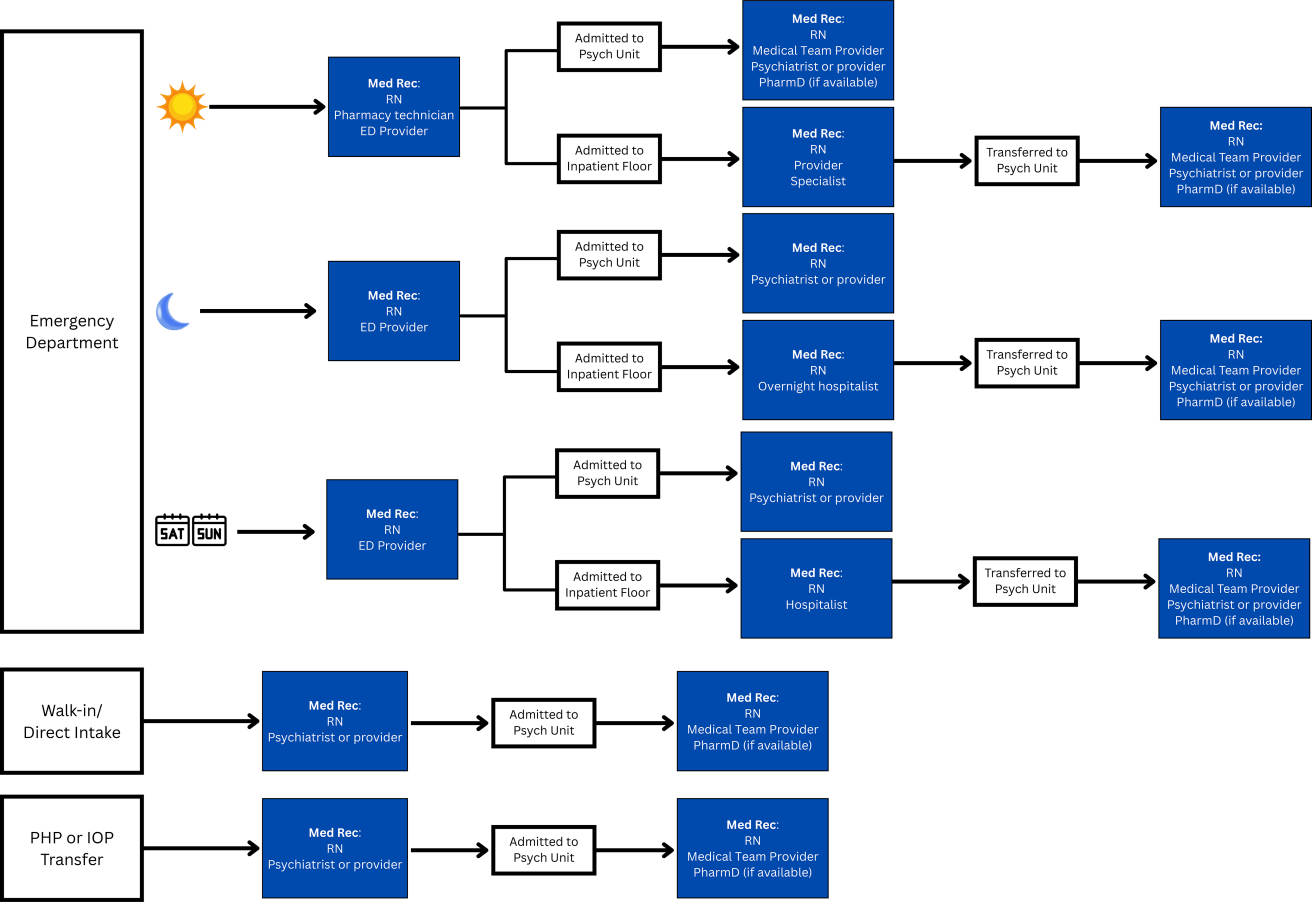
LAI-AP Medication Reconciliation Workflow Tracing Example; ED, Emergency Department; IOP, Intensive Outpatient Program; PHP, Partial Hospitalization Program.

**Figure 2:**
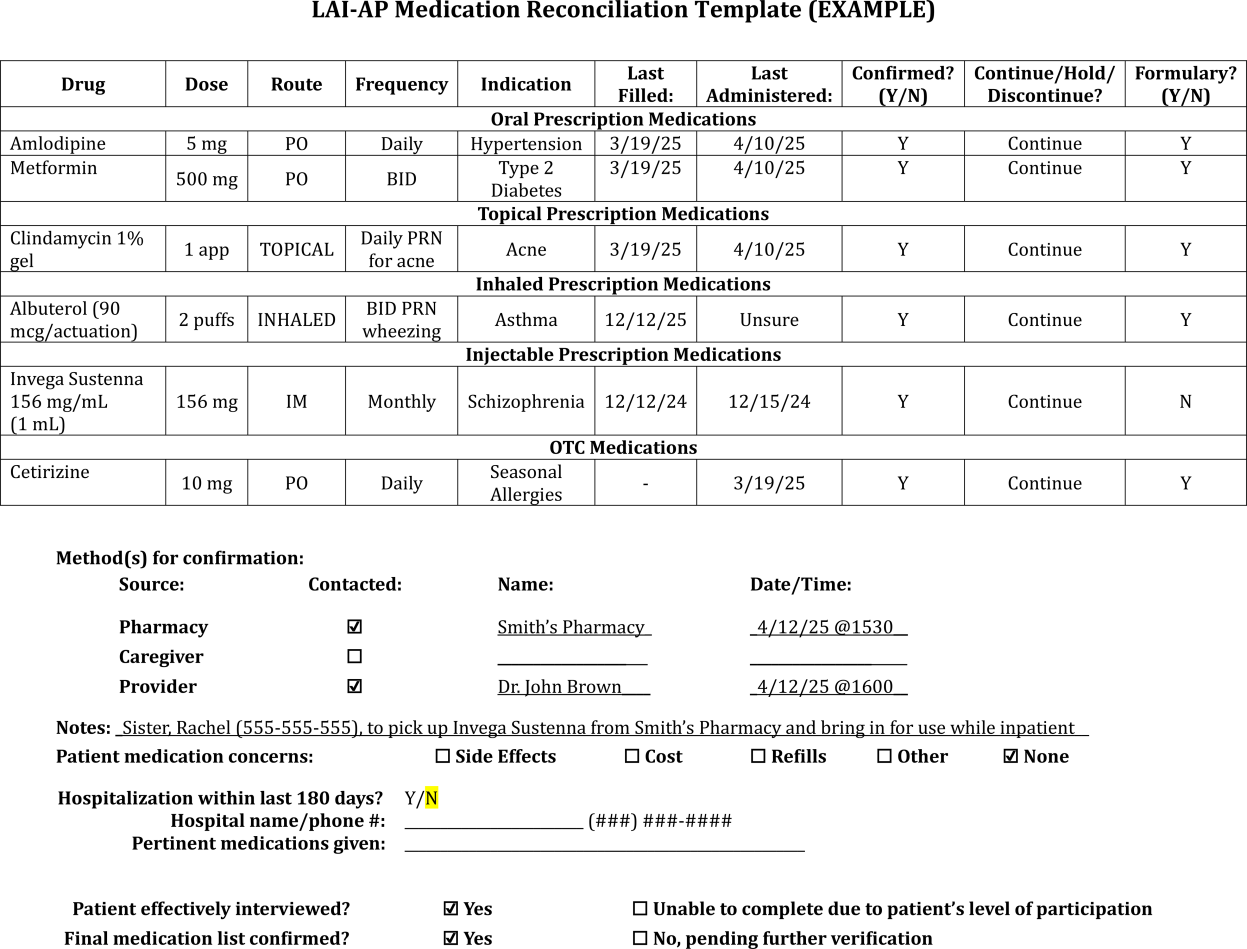
LAI-AP medication reconciliation template example.

- Create a flow chart describing current medication reconciliation practices (**Figure 1**).
  • Reduce unnecessary workloads through the identification of duplicated medication reconciliations conducted by multiple disciplines, especially those secondary to a lack of documentation, communication, or review.
  • For health systems encompassing different settings of care, map out various admission points to acknowledge vulnerable links where medication reconciliation enhancement can be focused (e.g. overnight admissions, weekend admissions, ED, walk-in intake, inpatient transfer, intensive outpatient or partial hospitalization program).
- Delegate and define roles for executing medication reconciliation process.
  • Ensure adequate staffing of medication reconciliation technicians and pharmacists.
  • In settings where pharmacist-led medication reconciliations are not feasible, success has been reported for nursing-led triage procedures following education on a standardized medication history directed by pharmacists (31).
  • Utilize interpreters to help obtain collateral where language barriers exist.
  • If the medical status of the patient, complexities, or inadequate staffing prevent a sufficient medication history from being completed, this should be clearly communicated and reconciled within 24 hours of admission.
- Create a standardized medication reconciliation checklist.
  • Standardize collateral questioning and documentation through a script or checklist.
  • Engage stakeholders and leaders from different disciplines to collaborate on what should be included in the checklist.
  • Promote inquiries about periodic injections, as these may be overlooked since these are not administered daily and are provided in a variety of healthcare settings.
  • Gather caregiver, point-of-contact, psychiatrist, and pharmacy information for collateral data as soon as the patient can convey this.
  • Contact secondary sources if the patient is a poor historian or to confirm information.
- Always ask the patient if they have recently (within at least the last 12 months) been seen at another hospital or emergency room and begin the process of requesting that hospital’s records (including the medication administration records) immediately to avoid delays.
- While EHR-integrated pharmacy claims data is a helpful resource, if an LAI-AP is listed in the patient’s claim history it is important to call the pharmacy, clinic, or hospital to confirm that the dose was picked up and if it was administered, making specific note of the date.
  • Because some LAI-APs may have longer dosing intervals, ensure the appropriate function is selected so that the claims report includes at least those in the last 180 days (about 6 months) or longer if possible.
- Document medication reconciliation completion and findings (**Figure 2**) in an easily accessible, centrally located section of the EHR to encourage review by other disciplines.
- For planned visits, encourage the patient to bring in a list or bottles/boxes of their current medications, being sure to ask about any injections since their last visit.
  • Consider the incorporation of a pre-registration medication reconciliation checklist into the pre-visit call while the patient has easy access to their medications and involvement of caregivers.
- Perform regular audits of admitted patients to check for documentation of completed medication reconciliations with special attention to those that may have received an LAI-AP.
  • If absent or insufficient medication reconciliations are identified during an audit, perform a root cause analysis to determine systematic and/or patient specific factors that may have contributed.
  • If there are time constraints for randomized auditing, consider identifying patients with risk factors for frequent admissions (e.g. RD patterns, extensive medication lists, low health-literacy, cognitive impairment) to concentrate on auditing efforts.

## LAI-AP medication use process

### Reasoning

#### Initiation or re-initiation

LAI-APs have varying loading doses, which may differ from maintenance doses, and/or oral overlap periods, also known as the specific time the oral formulation of the LAI-AP active ingredient should continue to be taken following the first injection of certain products in order to maintain therapeutic drug concentrations while the drug concentration contributed by the injection slowly builds up to a therapeutic level. Clinicians should be cognizant of the loading and oral overlap duration differences even among LAI-AP versions of the same oral molecule (e.g., Risperdal Consta and Uzedy, Abilify Maintena and Aristada). Errors associated with LAI-AP initiation may include (where applicable): an exclusion of one or more loading doses, providing dual loading doses, exclusion of maintenance doses, incorrect oral overlap durations, incorrect dosing based on kidney or liver function, incorrect conversions to the proper LAI-AP doses best corresponding to the oral dose the oral dose with which the patient was stabilized, and failing to ensure efficacy and tolerability of the oral antipsychotic prior to initiation of the corresponding LAI (32).

The standardization of order entry and implementation of computerized decision support systems within an EHR have been highlighted as key advancements across healthcare specialties by reducing medication errors and adverse drug events while also saving time (33–36). These order sets can guide providers to the correct LAI-AP initiation strategy and prompt placement of orders for all applicable loading doses and subsequent maintenance doses once selected (10). Entering maintenance dose orders during inpatient admission, even if the patient is unlikely to receive a maintenance dose during the admission, can help facilitate the generation of the LAI-AP prescription for the correct dose on the correct date during discharge reconciliation. Where feasible, clinicians should administer all LAI-AP loading doses and/or complete the entire oral overlap period prior to a patient’s discharge. Unpublished data demonstrate a significant reduction in 30-day risk of emergency psychiatric encounters following discharge from a psychiatric inpatient unit when both paliperidone palmitate doses were administered to an inpatient versus only one initiation dose. We thank J. Jones et al. (poster, April, 2023) for noting this ambiguity. Although computerized physician order entry (CPOE) systems have been underexplored in the psychiatry setting, a pre- and post- study found implementation of a pharmacist-supported CPOE system with an integrated clinical decision support system (CDSS) led to a 45% risk reduction of a drug-related problem (37).

Because of the prolonged half-life of LAI-APs, restarting the medication may not be as simple as just administering the last LAI-AP dose, depending on how long the patient has been nonadherent or if steady-state had been achieved prior to nonadherence (38). Sub- or supratherapeutic levels may occur if the incorrect LAI-AP loading sequence is restarted, or if an oral overlap is not correctly provided. Importantly, those who previously responded to a specific LAI-AP at a particular dose may fail to re-attain adequate response or remission on the same agent or at the same dose, based on the knowledge that schizophrenia may progressively worsen over time, particularly with subsequent relapse of psychotic episodes (39). As such, a formal assessment of the patient by a psychiatrist is highly recommended before restarting an LAI-AP, especially if the patient is currently psychiatrically unstable.

#### Patient education

One barrier identified in LAI-AP initiation is that most patients are unfamiliar with LAI-APs and may be ill-informed that this is a treatment option (40, 41). Most LAI-APs offered are used to address patient relapses instead of being used preemptively to prevent them although it has been shown that early-phase patients with schizophrenia had high acceptance rates of LAI-APs when offered (42, 43). During education regarding LAI-APs, it is important for clinicians to highlight the benefits of LAI-AP use and not focus solely on the modality (e.g. “shot”, or “injection”) (44).

Appropriate selection of an oral antipsychotic hinges on major factors such as the side effect profile, corresponding LAI-AP availability, medication expense, and patient preference. When selecting an initial agent, clinicians should attempt to broach the subject of an LAI-AP with the patient even at the very beginning of therapy. Education on the most common side effects should be provided alongside a few different oral options while making sure not to overwhelm the patient with information or options. Involving patients as part of a shared decision-making process may increase the likelihood of LAI-AP adherence, especially if patients do not immediately see efficacy or if side effects arise (45). Additionally, patients should be taught about the importance of continuing their medication even after their symptoms have abated. Recognition of barriers such as comprehension and cultural influences may help providers to better understand the patient’s point of view. These barriers may be mitigated to some extent by using printed materials, teach-back methods, and motivational interviewing to help patients better understand and feel more comfortable with trialing an LAI-AP. Still, it is often beneficial to involve patients’ families in this education, particularly when patient comprehension is lacking but also so they can help support the patient through this process. Documentation of this education can be considered a crucial part of patient care. Further, clinicians will meet legal and regulatory standards through adequate documentation of education.

#### Preparation

LAI-APs vary significantly with respect to how they should be prepared and administered. While clinicians may try to memorize all the distinct information for each shot, it is more useful and important for them to familiarize themselves with where to find information on preparation and administration. Because many LAI-APs are commonly packaged as pre-filled syringes, clinicians may errantly believe no extra preparation is required, when specific preparations such as syringe shaking may be needed to ensure solution homogenization prior to administration. Older LAI-APs (including haloperidol decanoate and fluphenazine decanoate) are supplied as multi-dose vials; the ISMP recommends these LAI-APs be drawn up by a pharmacist and dispensed as patient-specific doses where feasible (10). If a nurse has the responsibility of preparing an LAI-AP dose, the pharmacist should ensure that the full kit along with preparation instructions and required diluent (if not included within the kit) are provided to reduce the risk of an error occurring during this critical step.

#### Dispensing

Some distribution strategies have been developed to address significant costs associated with clinician-administered, high-dollar medications while still providing access to necessary treatment. Mark-up costs are attributed to the practice of buy-and-bill where the provider purchases and maintains inventory of medications, administers them in-house, and submits a claim for drug cost reimbursement. One method of bypassing the buy-and-bill practice is having providers receive the medication directly from a specialty pharmacy for administration, often referred to as “white bagging.” Another option used to bypass this, known as “brown bagging,” is when the patient picks up their medication from the pharmacy and brings it in with them to their appointment for administration. In this case, the responsibility of transportation and handling of the medication falls on the patient. Inherent issues with this method may be the uncertainty that the medication is stored in a suitable environment by the patient or has not been otherwise adulterated and that it is not wrongly administered by someone other than a healthcare provider. While white bagging and brown bagging have shown to reduce costs by large payers in some instances, interviews of providers revealed concern for failings of these practices related to poor transitional care coordination, disruptions due to treatment delays, and access challenges for vulnerable patient populations (46, 47). Legislation has been introduced in more than 20 states to address payer-mandated white-bagging including guardrails, bans, and prohibiting use (48).

“Gold bagging”, also referred to as “clear bagging” is when a provider or hospital uses its own internal specialty pharmacy to dispense and transport the medication to the site where it is to be administered. The drug cost involves the internal pharmacy instead of the provider directly. This presents the highest level of care as it provides real-time data documented within an integrated EHR. It also allows for impromptu dose or treatment changes during the current visit as the LAI-AP would be readily available and would not depend on delivery from an outside pharmacy or on the patient to retrieve the LAI-AP separately from an outside pharmacy.

#### Administration

Barcode medication scanning should be implemented to check the LAI-AP both before dispensing and administration of the medication to not only ensure the right medication for the right patient is being provided, but also to ensure EHR charting is done properly (10). Clinicians should frequently rotate the LAI-AP injection sites to avoid the risk of lipohypertrophy. Some LAI-APs come with multiple needle sizes to avoid accidental administration in subcutaneous tissue rather than intramuscularly for patients of a certain weight. In nearly all instances, the clinician should not use any other needles than the ones supplied by the manufacturer within the packaging. Other LAI-APs are required to be administered subcutaneously and form a palpable, semi-solid lump under the skin. Injecting a shot using an incorrect technique may result in improper release of the medication, including dose-dumping or accumulation or may lead to an injection site reaction such as cellulitis. Studies have shown that more than half of injections intended for the intramuscular site are actually errantly administered subcutaneously, with even higher rates in obese patients (49). Secondary to increased blood flow to the arm or thigh versus the abdomen or buttocks, higher maximum plasma concentrations of the LAI-AP can occur. Conversely, because of the slower release of the LAI-AP when injected in the gluteus, the half-life of the medication when administered here may be prolonged, resulting in more dose accumulation. In either instance there exists a possibility of side effects.

Pharmacists are authorized to administer LAI-APs in 48 states (50). These strides expand access to patients with serious mental illnesses across a variety of healthcare settings, especially in areas with mental health professional shortages (51, 52). The addition of this unique pathway for patient interaction provides alternate sites of care and reduces stigma but further accentuates the need for standardized documentation that can be easily communicated not only across different healthcare systems, but also sites that differ in usual patient care services (i.e. pharmacies, outpatient clinics, hospitals, and residential treatment centers). As mentioned, although pharmacy claims data may be available, these data are only partially reliable. With the ability for pharmacists to administer LAI-APs, there may be confusion whether the medication was dispensed vs dispensed and administered at the pharmacy.

#### Monitoring efficacy & tolerability

It is apparent that there is a vast complexity to antipsychotic responsiveness. Efficacy from LAI-APs is often defined on the basis of the likelihood of psychosis relapse or hospital readmission within studies; but relapse is only a secondary marker, as compared with the Brief Psychotic Rating Scale (BPRS), the Positive and Negative Symptom Scale (PANSS), or the Clinical Global Impression (CGI) scale, which are all primary markers. Studies have not consistently demonstrated greater reductions in these scales with LAI-APs over oral antipsychotics, and any potential reductions in these parameters later in therapy are likely secondary to increased adherence rates (53). This is further evidenced by the fact that real world, registry studies tend to more consistently demonstrate a benefit of LAI-APs over their corresponding oral formulations whereas randomized-controlled trials typically do not (54). Although time constraints often prevent the implementation of these scoring tools into clinical practice, their use may theoretically provide better insights into how a patient is progressing during treatment, and whether the patient has reached a point where the LAI-AP should be administered. This may be especially prudent where patients are seen by multiple providers who may not know how the patient was doing at baseline (i.e., prior to antipsychotic therapy).

Frequent assessments of adverse effects, including vitals, metabolic parameters, and extrapyramidal side effects, should be performed while the patient is on the initial oral antipsychotic and throughout LAI-AP therapy. Documenting any notable vital sign changes, especially those that may be concerning for neuroleptic malignant syndrome, or any movement disorders, may protect from the accidental initiation of a poorly tolerated LAI-AP resulting in potentially intractable adverse effects. While the intramuscular LAI-AP formulation of olanzapine requires specific, 3-hour monitoring by a healthcare professional for post-injection delirium and sedation, currently no other LAI-APs dictate this monitoring. However, case reports of fatalities possibly secondary to the LAI-AP have been reported (55). Clinicians should be knowledgeable of dose-dumping phenomena as well as anaphylaxis or other allergic reactions caused by LAI-APs which could result from excipients (56, 57).

The Drug Attitude Inventory (DAI) is a 10-item true/false questionnaire which may be used to predict treatment compliance in patients with schizophrenia, as well as the response to treatment with antipsychotics. Clinicians should consider the baseline use and documentation of the DAI scale score as a potential rationale for use of an LAI-AP, and periodically to assess for the risk that a patient may be considering withdrawing from treatment. Additionally, quality of life (QoL) should be maintained as an ongoing assessment for all patients where switching from an oral antipsychotic to an LAI-AP is being considered, to ensure patient-centered. In a 2023 systematic review, the authors determined QoL to be a central element for selecting the best treatment for patients with schizophrenia (58). Ascertaining patient buy-in through shared decision making when selecting an antipsychotic may be a critically important factor in improving adherence (59). Using various scales such as the Satisfaction With Life Scale or the Schizophrenia QoL Scale can objectively determine areas where intervention may be required (58). To this effect, clinicians should consider the ramifications of switching to an LAI-AP simply for the sake of doing so. The exorbitant costs of LAI-APs cannot be understated, with one study noting a lack of cost-effectiveness when oral paliperidone was switched to paliperidone palmitate (i.e. Invega Sustenna, Trinza, or Hafyera) for patients with schizophrenia who are currently stable (60).

A growing amount of literature supports therapeutic drug monitoring (TDM) for antipsychotics, with newer guidelines providing recommended levels for various antipsychotics (61). While the pharmacokinetics of LAI-APs differ from oral antipsychotics and overall literature of TDM for LAI-APs is sparse, TDM can still provide many theoretical benefits (62). When patients are psychiatrically stable, routine TDM provides a baseline assessment and sample for comparison if the patient later destabilizes. In instances of presumed toxicity, such as dose-dumping, TDM may be especially important. Although TDM may be more applicable to oral medications from an adherence standpoint, TDM may still prove useful for those taking LAI-APs. Further, because many manufacturers determined therapeutic equivalences for LAI-APs from pharmacokinetic modeling and simulated data rather than actual TDM, current dosing strategies may not provide comparable levels to their oral counterparts, especially considering interindividual variabilities (62, 63). Clinicians must also be very cognizant of the timing of the last LAI-AP to ensure they are not making inferences from an incorrectly drawn level.

#### LAI-AP switching or discontinuation

Another important consideration prior to initiating LAI-AP is ensuring an adequate trial of the corresponding oral agent. In particular, trialing oral antipsychotic for a long enough period (at least five half-lives) may help to prevent some concerning adverse effects. Where a patient has failed to respond to or has significant intolerance to an LAI-AP, documentation of the length of the corresponding oral antipsychotic trial prior to initiation of the LAI-AP may provide a rationale behind these complications. Because many maintenance LAI-AP doses are based on the corresponding oral daily dose necessary to attain comparable serum concentrations, clinicians should also document the specific dose of oral antipsychotic the patient was stabilized on prior to initiation of the LAI-AP (64).

A retrospective chart review analyzing antipsychotic treatment changes showed oral second-generation antipsychotics (SGAs) were switched to LAI-APs in almost 11% of cases due to nonadherence while SGA LAI-APs were switched to SGA oral antipsychotics in 11% of cases because of intolerability or inefficacy (65). One in five of these switches were not appropriately documented in prescriber notes. Even when switching among the same LAI-AP product with different administration frequencies thorough documentation is paramount, secondary to the risk of intolerability or inefficacy (e.g., stability on Invega Sustenna for at least four months is required prior to initiating Invega Trinza). There is a paucity of data regarding recommendations for switching from an LAI-AP to an oral antipsychotic, or from one LAI-AP to another LAI-AP (66). Still, knowing the date of the last injection is key. Clinicians often simply stop the previous LAI-AP and start another one at the next dosing interval. However, the previously administered LAI-AP will still be present in significant serum concentrations, so this tactic may be unfit for some patients.

When discontinuing the previously administered LAI-AP it is important to document the reason for discontinuation and the stop date of that LAI-AP within the notes so that clinicians can reflect upon and make informed dosing decisions.

### Best practice

**Figure 3:**
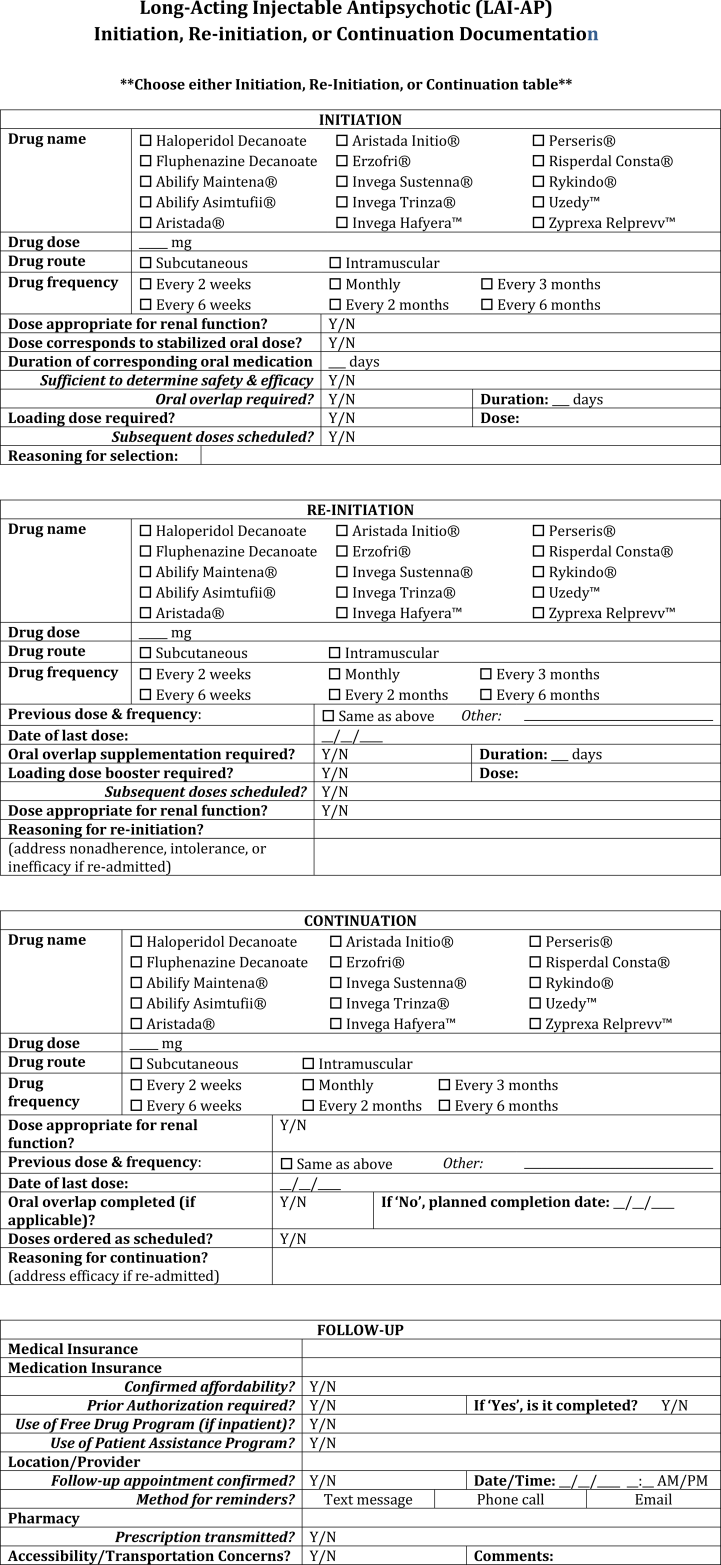
LAI-AP initiation, re-initiation, or continuation documentation template.

**Figure 4:**
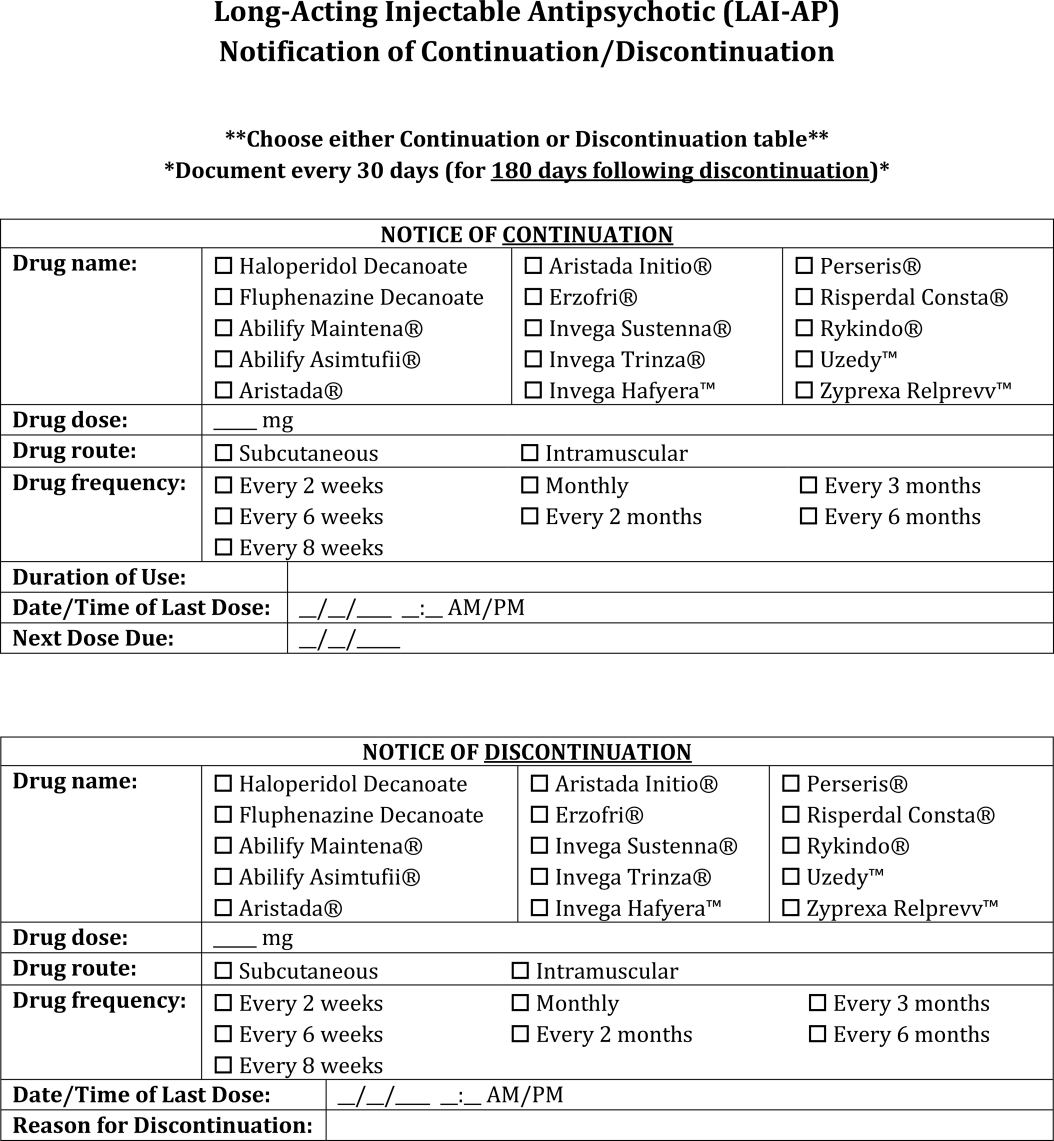
LAI-AP notification of continuation or discontinuation documentation template.

**Figure 5:**
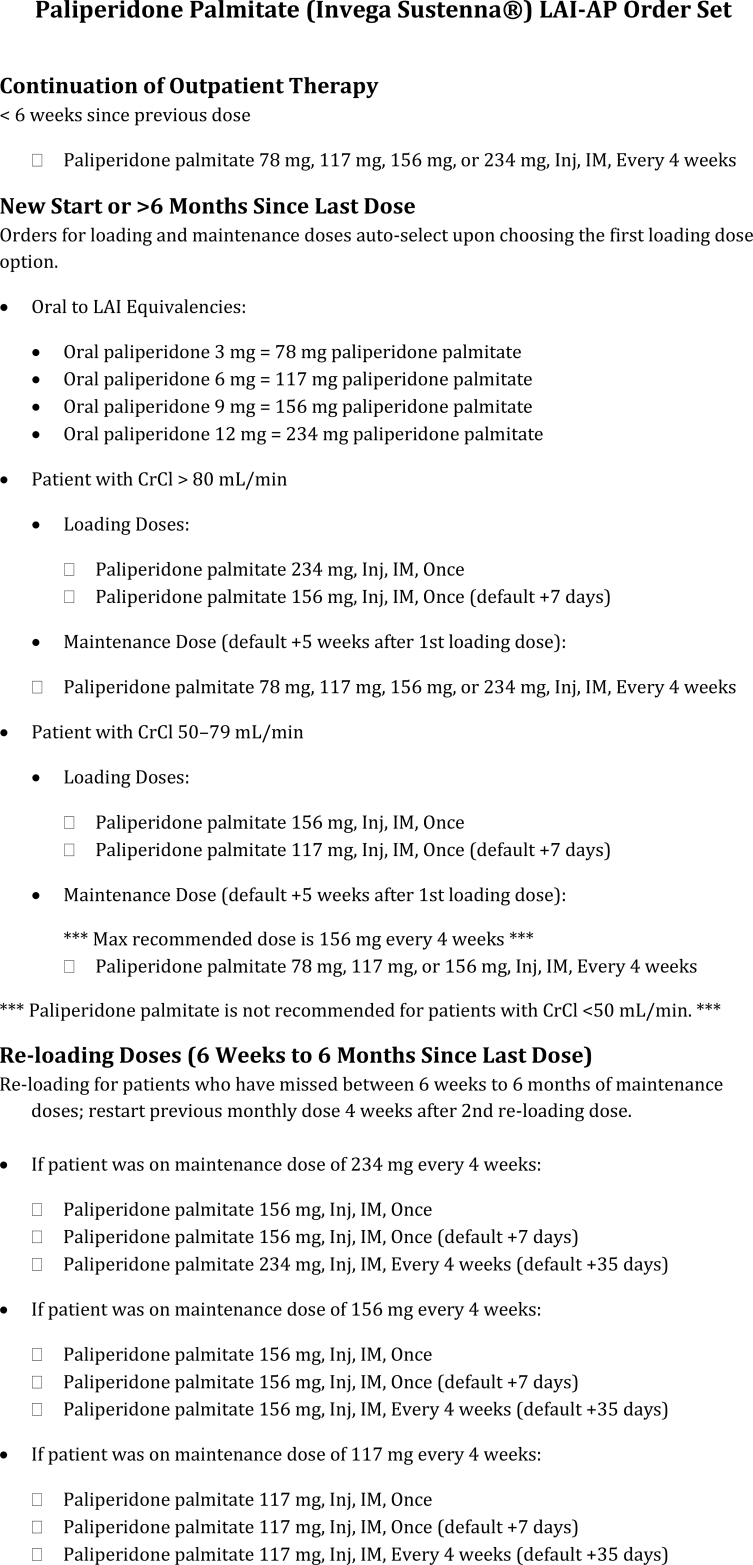
Paliperidone palmitate LAI-AP order set example.

**Figure 6:**
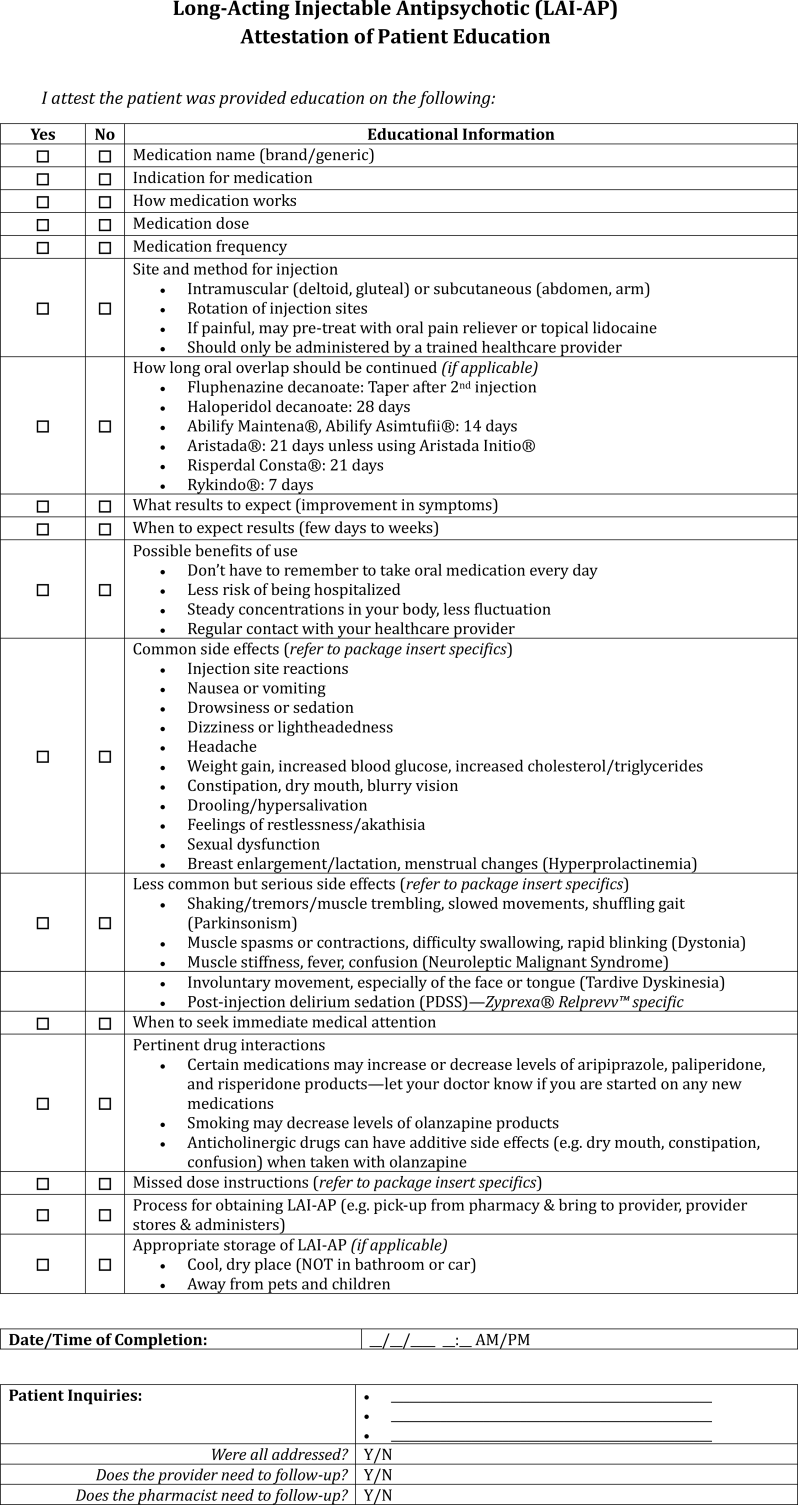
LAI-AP attestation of patient education documentation template.

**Figure 7:**
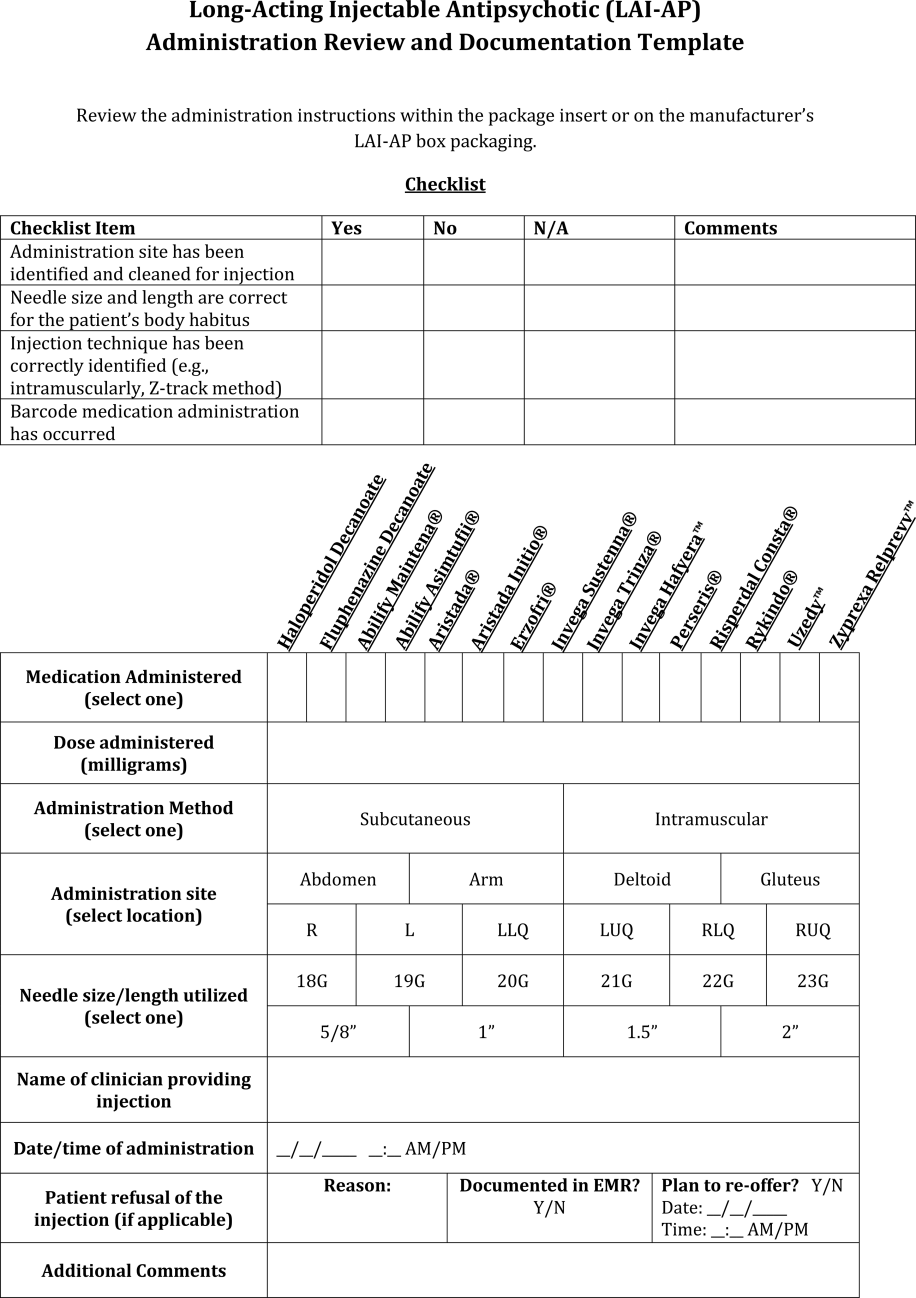
LAI-AP administration review and documentation template. L, Left; LLQ, Left Lower Quadrant; LUQ, Left Upper Quadrant; R, Right; RLQ, Right Lower Quadrant; RUQ, Right Upper Quadrant.

**Figure 8:**
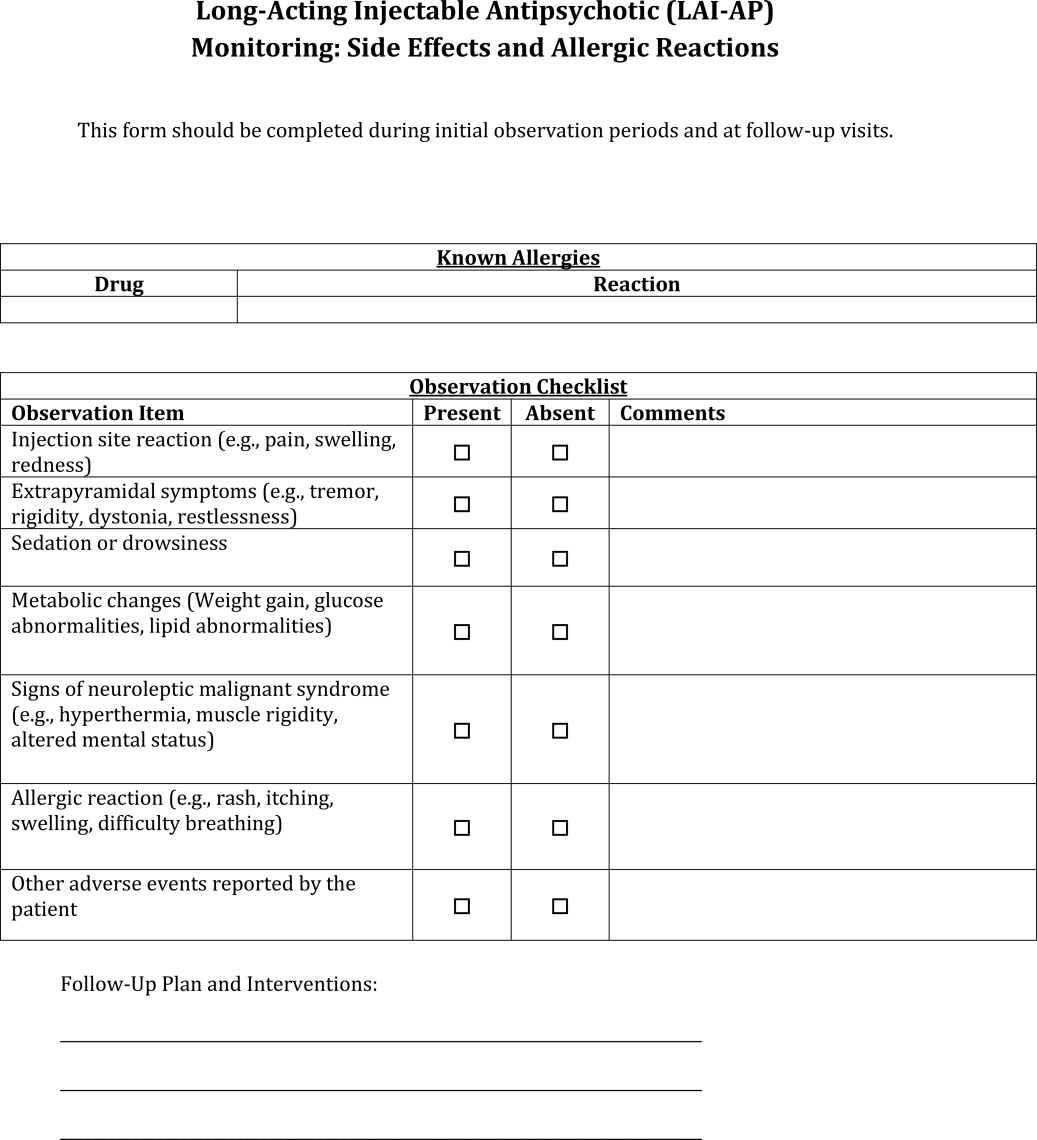
LAI-AP side effects and allergic reactions monitoring documentation template.

- Prior to Ordering:
  • Require last dose verification (practitioners must document when the last dose was administered before subsequent doses are ordered). Consider oral or short-acting injectable agents until this is verified.
  • Consider performing a QoL scale and baseline DAI scale to help establish additional rationales for initiation of an LAI-AP.
- Ordering LAI-APs during hospitalization (Inpatient):
  • For patients on medical units, consultation of a psychiatrist is highly advised prior to LAI-AP initiation, re-initiation, or continuation, predominantly when the patient is psychiatrically unstable.
  • Document the patient’s symptomatic improvement as well as the dose and duration of the oral antipsychotic medication provided prior to initiation of the corresponding LAI-AP (**Figure 3**).
  • Conduct regular chart reviews to ensure clinical reasoning for switching from an LAI-AP to an oral antipsychotic or vice versa is documented appropriately for reference in future treatment. Leaving out pertinent information about intolerability or inefficacy could delay effective treatment and prolong hospitalization.
  • Continue to document the stop date of the LAI-AP within the chart for at least a period of 6 months after discontinuation as this period of time is likely to influence the re-initiation dosing of LAI-AP agents (**Figure 4**).
  • Utilize comprehensive LAI-AP order sets which include information on appropriate oral overlap sequences (where applicable), appropriate LAI-AP initiation dose(s) and maintenance dose based on stabilizing oral dose, dosage adjustments for renal dysfunction, and what to do in the instance of a missed dose (**Figure 5**).
  • Provide all initiation doses of an LAI-AP prior to the patient discharging from the hospital to reduce risk of patient relapse/readmission, where applicable.
  • Use scheduled dosing frequencies of LAI-APs based on their respective dosing intervals instead of single dose administrations when entering or verifying medication orders.
  • Note the number of LAI-AP maintenance doses the patient has received, as this can assist with knowing whether the patient is at steady state on a particular dose.
- Prescribing of LAI-APs upon discharge (Inpatient):
  • Generate a prescription with the specific LAI-AP, dose, dosing frequency, and date that the next injection is required directly from the inpatient medication order.
- Prescribing of LAI-APs (Outpatient):
  • Ensure reasoning for selection, initiation, continuation, or re-initiation is clearly documented and easily accessible for other clinicians to review.
    • Development of documentation templatesmay help save time and encourage comprehensiveness (**Figure 3**).
    • Include considerations regarding allergies, tolerability, and previous efficacy.
    • For instances of switching between LAI-APs or discontinuation, continue to document the date and time of the last dose administration for at least 6 months following discontinuation (**Figure 4**).
    • Conduct regular chart reviews to support appropriate documentation practices.
  • If the LAI-AP is a continuation of an already initiated product, verify and document confirmation of when the last dose was administered (**Figure 3**).
  • Address person-specific barriers to LAI-AP affordability and access.
    • Insurance coverage, patient assistance, and out-of-pocket costs should be confirmed and communicated as part of the shared decision-making process.
    • If payer models require a “brown bagging” process, verify the patient has adequate transportation resources to pick up medication and can arrive to their scheduled appointment for administration at the frequency required by the selected LAI-AP.
    • For LAI-APs requiring loading doses, verify the feasibility of procuring all loading doses within the recommended time frame (e.g. Invega Sustenna 234 mg treatment on Day 1 and 156 mg between Days 4 and 12). Having both loading dose prescriptions sent to a pharmacy for pick-up or delivery at the same time may reduce potential for transportation issues and therefore missed doses.
- Providing Patient Education.
  • Inform patients with approved indications that LAI-APs are an available treatment option and discuss individualized pros and cons as part of a shared decision-making process.
  • Allow the patient to have buy-in for selection of the LAI-AP when possible and feasible.
  • Provide written and verbal information regarding side effects to the patient.
  • Document any education or answers to specific inquiries provided to the patient within the EHR (**Figure 6**).
    • Creation of a standardized template within the EHR that notes all the specific LAI-AP education components on which the patient was educated can streamline the process and save time.
- During Preparation:
  • Review the package insert or manufacturer’s website for specific preparation instructions.
  • Require second verification of prepared doses prior to administration.
  • Utilize barcode scanning to double-check the appropriate formulation and any diluent necessary for preparation.
  • Record the manufacturer’s lot number for reference in the instance of an adverse effect related to the LAI-AP.
- During Dispensing:
  • Dispense patient-specific doses whenever possible.
  • Always provide the package insert along with the prepared injection or provide specific information on how to inject the medication.
  • Gold Bagging should be the standard health system practice whenever possible as storage, dispensing, and administration would all happen internally.
- During Administration:
  • Review the package insert or manufacturer’s website for instructions on how and where (e.g. deltoid versus gluteal) to administer the LAI-AP.
  • Rotate injection sites frequently.
  • Require documentation of (**Figure 7**):
    • Injection administration site.
    • Injection technique (i.e., subcutaneously, intramuscularly, or Z-track method).
    • Needle size and length (e.g. 21G, 1.5 inches).
  • Utilize barcode scanning of the prepared product and patient identifiers.
  • If a patient refuses administration, transfers to another unit, or another situation arises where the dose is not given, it’s important the nurse documents “dose not given” within the EHR so that this dose is not accidentally omitted or assumed to have been administered.
- Perform and note baseline PANSS, BPRS, or CGI-S scores prior to antipsychotic therapy, on at least a weekly basis during hospital admissions, and prior to initiation of an LAI-AP.
  • Establish efficacy of the corresponding oral antipsychotic for a period of up to 4 weeks prior to initiation of the LAI-AP where feasible.
    • Where patients are refusing oral therapy, consider the use of a short-acting injectable antipsychotics to either motivate the patient to consider taking the oral medication or to trial efficacy and safety prior to initiation of the LAI-AP.
    • If a patient on an LAI-AP remains psychiatrically unstable, trialing an addition of the corresponding oral antipsychotic is recommended before increasing the LAI-AP dose or frequency. TDM may also be of benefit in these situations.
- Obtain all relevant baseline laboratories and document any notable movement disorders prior to starting the LAI-AP.
  • Establish tolerability of the corresponding oral antipsychotic for a period of up to 4 weeks prior to initiation of the LAI-AP where feasible.
  • Upon starting the LAI-AP, perform every 3 to 6-month assessments of the Abnormal Involuntary Movement Scale (AIMS), and document these within the EHR.
- Monitor patients for at least 15–30 minutes after their first and second doses of the LAI-AP due to the potential for anaphylaxis or other allergic reactions. Document any allergic reactions within the EHR. Many EHR systems have drug-allergy interaction checkers which may help capture and prevent future allergic reactions.
  • Where allergy or side effect documentation is concerned, clinicians should provide as much explicit detail as possible to avoid confusion. The creation of a standardized LAI-AP allergy/side effect template (**Figure 8**) may help facilitate this process (67).
- Consider the use of TDM for patients who display LAI-AP treatment inefficacy or intolerability.
  • Document TDM levels in an easily accessible location, noting the LAI-AP dose and the time the level was drawn (ex: trough).

## Transitions of care and discharge planning

### Reasoning

The World Health Organization reported up to 80% of patients had at least one medication discrepancy upon hospital discharge (19). Lack of healthcare system interoperability contributes to segmentation in healthcare provision which increases the risk for medication errors related to duplication or omission, especially in behavioral health settings (27). Drug omission is a frequent error occurring at discharge and may result in missed doses in the outpatient setting, which increases the patient’s risk for relapse and readmission (68). With limited literature on LAI-AP documentation, one unpublished study comparing one-time vs. scheduled LAI-AP doses and discharge medication reconciliations on both psychiatric and nonpsychiatric units found that scheduled doses of LAI-APs were more frequently continued on the discharge medication reconciliation with a specified due date at discharge. We thank M. Farah et al. (poster, April, 2023) for noting this ambiguity.

The revolving door (RD) phenomenon describes patients with frequent readmissions to behavioral health units. By nature, individuals experiencing the RD phenomenon are more likely to engage with health systems frequently that lack interoperability. Even when returning to the same facility, the absence of thorough documentation—such as prior treatments and discharge plans—can contribute to this cycle of readmissions, as well as medication errors or delays in therapeutic treatment due to discontinuity (69).

Social and demographic risk factors associated with higher rates of RD hospitalizations include being single, unemployed, younger patients between the ages of 15–35 years old, and those with less education (70). Recognizing these factors can assist in identifying individuals where comprehensive, standardized documentation efforts may be especially beneficial with the intent of enhanced care continuity and communication between settings (71–73). Additionally, co-occurring serious mental illness (SMI) and substance related disorders, medical comorbid conditions, longer hospital length of stays, commitment to community residential facilities, and LAI-AP FGA prescription at discharge have been associated with RD hospitalizations. With data supporting increased LAI-AP use in younger individuals experiencing FEP, real-world predictors of rehospitalization in this population present valuable targets for improving clinical documentation efforts and reinforcing continuity of care through more effective follow-up strategies (3).

The identified relation of an LAI-AP prescription at discharge to RD patterns may be more attributable to the nonadherence characteristic of patients for which these medications are recommended. However, it is important to note that while LAI-APs have been shown to assist with medication adherence, they do not guarantee adherence, and patients may still discontinue their LAI-AP even after appropriate follow-up planning (13). It is worth also noting though that LAI-AP have been shown to significantly extend the time to psychosis relapse relative to oral antipsychotics following random discontinuation, but this was seen in patients who had been stabilized on antipsychotics for half a year on average prior to discontinuation (74). Thus, a key consideration if an LAI-AP is initiated during an inpatient admission is that all efforts should be made to ensure its continuation upon discharge.

Studies looking at the RD phenomenon tend to be more focused on the patient and patient-related risk factors as opposed to potential contributing systematic factors in the transitions of care processes worth assessing (70). Notably, patients who do not follow-up after discharge are more likely to experience revolving door patterns (75). One study focused on successful discharge planning found that over 20% of patients on a psychiatric inpatient unit were not scheduled an outpatient follow-up appointment, even though this is considered a standard of care by national organizations (76). The probability of patients with mental health admissions to attend a mental health outpatient follow-up appointment within the first 7 days post-discharge was nearly doubled when inpatient staff scheduled the appointment for the patient as part of discharge planning, underscoring the importance of process development within transitions of care. Documentation of this key step may offer confirmation of completion and closed loop communication to other providers.

Further, interdisciplinary treatment team meetings are an essential component of psychiatric care wherein members discuss the patient’s progress, current treatment, and facilitate follow-up care for the patient upon discharge from an inpatient stay (77). These collaborative meetings commonly occur weekly and often include a psychiatrist, nurse, social worker, and occupational therapist. Data are increasingly supportive of integration of clinical pharmacists into these meetings, with advocates reporting significant benefits through reductions in medication-related errors. Early communication regarding the discharge plan including transitioning a patient to an LAI-AP may prevent delays in discharge as well as prevent the patient from returning due to an adverse drug event as can happen without adequate demonstration of corresponding oral agent tolerability or inability to continue the medication outside the hospital due to issues around affordability, for instance. This was demonstrated within an unpublished work and we thank M. Cervenak et al. (poster, April, 2024) for noting this ambiguity. Unfortunately, LAI-APs are frequently associated with higher drug costs, and standard health insurance and Medicaid plans may not fully cover the entirety of the LAI-AP costs, leaving some patients with significant co-pays (78, 79). Patient assistance programs, while available for many LAI-APs often only cover those who are indigent and without health insurance, not patients who have insurance that does not fully cover the costs. Thus, affordability of an LAI-AP must be considered prior to initiation during an inpatient admission. Ultimately, implementing effective strategies for clear, shared documentation and communication when discharge planning can facilitate a patient’s successful transition from acute care mental health care to the community (80).

While variability exists regarding the setting in which an LAI-AP should be first initiated, a consensus exists around telephone and text messages reminders as strategies for improving patients’ adherence to LAI-AP administration appointments (32, 81). Timing of follow-up psychiatric appointments in the outpatient setting should be aligned whenever possible with maintenance LAI-AP frequency requirements (32). Ideally, offering LAI-AP administration by a mental healthcare professional in a patient’s home, as well as involvement of family members, peer specialists, or counselors in a patient’s care would also support appointment adherence (81). Additionally, arranging for patient travel or for home health to receive the injection is a crucial component.

### Best practice

- Inpatient Setting:
  • During an inpatient transfer, relay the last LAI-AP dose administered and confirm it is documented in the medication administration record.
  • Ensure affordability or enrollment in a patient assistance program for the LAI-AP prior to initiating a patient on the medication to help prevent outpatient delays, omissions in therapy, or having to switch medications after the LAI-AP has already been administered.
  • If the patient is to be transferred to another hospital, consider that hospital’s formulary or access to the LAI-AP prior to initiation.
  • Perform Activities of Daily Living (ADL) assessments on patients to ensure the highest level of care is sought for discharge.
  • Perform structured, interdisciplinary team transitions of care rounds weekly, including a pharmacist and social worker, with careful consideration of medication management and likely outpatient follow-up.
  • Always consider the patient’s ability to travel. Consider less frequent LAI-AP administrations for those patients or family members who are unable to commute more frequently or must commute longer distances.
    • Consider sending the LAI-AP to a community pharmacy which might be able to provide the injection.
- Upon Discharge from an Inpatient Stay:
  • Inpatient pharmacists should review discharge reconciliations for appropriateness.
  • Provide written documentation of the medication list to patient, caregiver, outpatient pharmacist, and outpatient psychiatrist detailing the LAI-AP dose, frequency, and route (noting equal importance of documenting LAI-APs that the patient *should no longer be taking*).
  • Verbally educate patients and caregivers.
  • Provide reminders and document notifications.
- Outpatient Setting.
  • Connect with behavioral health interprofessional teams throughout the different health systems in your area to foster open communication and shared documentation strategies.
  • For every new patient recently discharged from an inpatient facility, contact the facility and clarify any unclear LAI-AP administrations on the patient’s EHR from their stay, including one-time doses.
  • Review newly prescribed LAI-APs that are to be continued in the outpatient setting to confirm insurance coverage and affordability.
  • Proactively review LAI-AP prescriptions and contact the patients’ community pharmacies to verify that prior authorizations have been effectively submitted before a patient goes to pick them up.
  • If patients do not show up for their appointments, reach out to contacts for collateral information to determine if a patient has been admitted to an inpatient facility.
  • Communicate with patients and inpatient providers prior to discharge to promote a timely transition of care for patients to follow-up.
  • Reach out to the patient after discharge via text message or telephone call to remind them about their upcoming appointments.
  • Schedule visits whenever possible to align with the frequency of the prescribed LAI-AP administration due dates.
  • Develop behavioral health LAI-AP home visit programs to offer administration within the patient’s home or utilize home health services to reduce barriers to transportation.
    • Consider sending the LAI-AP to a community pharmacy which might be able to provide the injection.
  • Provide the patient, their caregiver, and other providers an ongoing list of the patient’s current medications and instructions, including the date and time of the most recent administration of their LAI-AP, how long they should continue taking the oral antipsychotic while the LAI-AP gets to steady state if pertinent, and any discontinued antipsychotics with rationale.

## Potential for innovative practices

Development of a registry or drug monitoring database specifically for documentation of LAI-AP dispensing and administration across all healthcare settings could serve as a workaround link to address the lack of interoperability between EHRs. While adoption of such a registry may start as individualized submissions by providers separate from current processes that mirror websites like those that have existed for the previous Clozapine Risk Evaluation and Mitigation Strategy (REMS) program, over time this registry may become more easily integrated into EHRs and automatically updated in real time similar to immunization information systems (IIS) and controlled substance prescription drug monitoring programs (PDMPs). This database would allow providers to gain a clearer understanding of a patient’s history with LAI-AP use and confirm recent administrations, hopefully leading to a reduction in duplicate administrations, continuation of necessary LAI-AP treatment, rotation of administration sites, and precautions regarding missed dosing titrations. Limitations to the implementation of such a database include a fragmentation of current health information exchange and strict laws protecting patient health information sharing such as the Health Insurance Portability and Accountability Act (HIPAA) (82). Overarching, policy-level solutions are paramount to overcoming these structural hurdles.

Adoption of a national EHR may facilitate the transfer of data more readily, saving time, effort, and money, and preventing the idiomatic “reinvention of the wheel” scenario wherein past failed trials of a medication are potentially perilously re-trialed (83). The national EHR system within Denmark, which integrated shared medication records, was noted to be subjectively beneficial to physicians, and a mutual-access EHR displayed greater communication and medication continuity versus those without mutual-access (84, 85).

Certain EHRs contain discharge acuity scoring tools based on specific patient parameters; these systems can help allocate resources to patients who are at high risk of readmission or relapse. Future uses of artificial intelligence and Bayesian modeling may help identify patients at high-risk for LAI-AP medication errors, including parameters such as having multiple comorbidities or the use of multiple pharmacies, multiple prescribers, or multiple antipsychotics including one or more LAI-APs. Such integrated programs could assist with performing comprehensive chart searches to determine the last administered LAI-AP dose, date, and location, providing a concise document on this information to which the clinician can refer, saving time and effort.

Some EHRs allow the embedding of web access links directly within specific medication orders or order sets. To mitigate the risks of inaccurate LAI-AP reconstitution or administration, consider the inclusion of Instructions for Use videos or guides provided by the manufacturer within the EHR for quick reference.

A smartphone application which is specific to LAI-APs may fulfill multiple purposes, not limited to injection reminders, but also may allow those administering the injection to transmit information about the administration directly to the patient’s application. This information could further be readily accessible among all the patient’s providers for clarity.

While this article aims to address existing gaps in LAI-AP safety practices through comprehensive documentation and draw guidance from generalities regarding medication safety, the limited reporting of medication errors and the sparse existing LAI-AP literature underscore the need for further development of treatment protocols, as well as encouragement of LAI-AP medication error reporting.

## References

[1] Salgueiro M Segarra R. Long-acting injectable second-generation antipsychotics in first-episode psychosis: A narrative review. Int Clin Psychopharmacol. (2019) 34:51–6. doi: 10.1097/YIC.0000000000000249, PMID: 30540617

[2] Tiihonen J Haukka J Taylor M Haddad PM Patel MX Korhonen P. A nationwide cohort study of oral and depot antipsychotics after first hospitalization for schizophrenia. Am J Psychiatry. (2011) 168:603–9. doi: 10.1176/appi.ajp.2011.10081224, PMID: 21362741

[3] Besana F Civardi SC Mazzoni F Carnevale Miacca G Arienti V Rocchetti M. Predictors of readmission in young adults with first-episode psychosis: A multicentric retrospective study with a 12-month follow-up. Clin Pract. (2024) 14:1234–44. doi: 10.3390/clinpract14040099, PMID: 39051293 PMC11270315

[4] Martiadis V Pessina E Raffone F Martini A Di Vincenzo M Della Rocca B. Efficacy and safety of adjunctive aripiprazole lai or paliperidone lai for the management of patients suffering from bipolar I disorder with comorbid obsessive-compulsive disorder. J Clin Med. (2025) 14(3):954. doi: 10.3390/jcm14030954, PMID: 39941625 PMC11818892

[5] Cipolla S Catapano P D’Amico D Monda R Sallusto NP Perris F. Combination of two long-acting antipsychotics in schizophrenia spectrum disorders: A systematic review. Brain Sci. (2024) 14(5):433. doi: 10.3390/brainsci14050433, PMID: 38790412 PMC11117856

[6] Yatham LN Kennedy SH Parikh SV Schaffer A Bond DJ Frey BN. Canadian network for mood and anxiety treatments (Canmat) and international society for bipolar disorders (Isbd) 2018 guidelines for the management of patients with bipolar disorder. Bipolar Disord. (2018) 20:97–170. doi: 10.1111/bdi.12609, PMID: 29536616 PMC5947163

[7] Borja VA Galbraith K. Medication-related issues associated with the documentation and administration of long-acting injectable antipsychotics. Int J Clin Pharm. (2019) 41:623–9. doi: 10.1007/s11096-019-00814-6, PMID: 30945046

[8] Braiki R Douville F Gagnon MP. Factors Influencing the Reporting of Medication Errors and near Misses among Nurses: A Systematic Mixed Methods Review. Int J Nurs Pract. (2024) 30:e13299. doi: 10.1111/ijn.13299, PMID: 39225448 PMC11608931

[9] Alshehri GH Keers RN Ashcroft DM. Frequency and nature of medication errors and adverse drug events in mental health hospitals: A systematic review. Drug Saf. (2017) 40:871–86. doi: 10.1007/s40264-017-0557-7, PMID: 28776179

[10] Institute for Safe Medication Practices. Ismp safety issues with long-acting injectable (Lai) antipsychotics. ISMP Med Saf Alert! Acute Care. (2023) 28:1–6.

[11] Keers RN Williams SD Vattakatuchery JJ Brown P Miller J Prescott L. Prevalence, nature and predictors of prescribing errors in mental health hospitals: A prospective multicentre study. BMJ Open. (2014) 4:e006084. doi: 10.1136/bmjopen-2014-006084, PMID: 25273813 PMC4185335

[12] Lebas R Calvet B SChadler L Preux PM Laroche ML. Relationships between medications used in a mental health hospital and types of medication errors: A cross-sectional study over an 8-year period. Res Soc Adm Pharm. (2024) 20:597–604. doi: 10.1016/j.sapharm.2024.03.006, PMID: 38531707

[13] Ross C Adams KS Crouse EL. Transitions of care: assessment of adherence to long-acting injectable antipsychotic treatment following discharge from inpatient psychiatry. Ment Health Clin. (2025) 15:9–16. doi: 10.9740/mhc.2025.02.009, PMID: 39974755 PMC11835369

[14] Fitzgerald RJ. Medication errors: the importance of an accurate drug history. Br J Clin Pharmacol. (2009) 67:671–5. doi: 10.1111/j.1365-2125.2009.03424.x, PMID: 19594536 PMC2723207

[15] Gleason KM Brake H Agramonte V Perfetti C. Medications at Transitions and Clinical Handoffs (Match) Toolkit for Medication Reconciliation. Rockville, MD: Agency for Healthcare Research and Quality (2012). Available online at: https://www.ahrq.gov/sites/default/files/publications/files/match.pdf (Accessed June 30, 2025).

[16] World Health Organization. The High5s Project – Standard Operating Protocol for Medication Reconciliation (2014). Available online at: https://cdn.who.int/media/docs/default-source/patient-safety/high5s/h5s-sop.pdf?sfvrsn=594d8e49_4 (Accessed June 30, 2025).

[17] The Joint Commission. Quick Safety Issue 26: Transitions of Care: Managing Medications (Updated April 2022) (2016). Available online at: https://www.jointcommission.org/resources/news-and-multimedia/newsletters/newsletters/quick-safety/quick-safety-issue-26-transitions-of-care-managing-medications/ (Accessed June 30, 2025).

[18] Woodyard A Ramdeo P Herzik KD Desselle SP. Reconciling medications in mental health assessments protects patients. Pharm Times. (2022) 88.

[19] World Health Organization. Medication Safety in Transitions of Care (2019). Available online at: https://iris.who.int/bitstream/handle/10665/325453/WHO-UHC-SDS-2019.9-eng.pdf?sequence=1 (Accessed June 30, 2025).

[20] Accomando M DeWitt K Porter B. Pharmacist impact on medication reconciliation of behavioral health patients boarding in the emergency department. Ment Health Clin. (2022) 12:187–92. doi: 10.9740/mhc.2022.06.187, PMID: 35801158 PMC9190267

[21] Mekonnen AB McLachlan AJ Brien JA. Effectiveness of pharmacist-led medication reconciliation programmes on clinical outcomes at hospital transitions: A systematic review and meta-analysis. BMJ Open. (2016) 6:e010003. doi: 10.1136/bmjopen-2015-010003, PMID: 26908524 PMC4769405

[22] Schnipper JL. Medication reconciliation-too much or not enough? JAMA Netw Open. (2021) 4:e2125272. doi: 10.1001/jamanetworkopen.2021.25272, PMID: 34529070

[23] Patel S Mathis AS Costello J Ghin HL Fahim G. Satisfaction with medication reconciliation completed by pharmacy technicians in an emergency department. P T. (2018) 43:423–8., PMID: 30013300 PMC6027853

[24] Hammour KA Farha RA Basheti I. Hospital pharmacy medication reconciliation practice in Jordan: perceptions and barriers. J Eval Clin Pract. (2016) 22:932–7. doi: 10.1111/jep.12565, PMID: 27198470

[25] Kennelty KA Chewning B Wise M Kind A Roberts T Kreling D. Barriers and facilitators of medication reconciliation processes for recently discharged patients from community pharmacists’ Perspectives. Res Soc Adm Pharm. (2015) 11:517–30. doi: 10.1016/j.sapharm.2014.10.008, PMID: 25586885 PMC4409924

[26] Degan TJ Kelly PJ Robinson LD Deane FP Wolstencroft K Turut S. Health literacy in people living with mental illness: A latent profile analysis. Psychiatry Res. (2019) 280:112499. doi: 10.1016/j.psychres.2019.112499, PMID: 31398576

[27] Lertxundi U Corcóstegui B Prieto M Gonzalez U Arana A. Medication reconciliation in psychiatric hospitals: some reflections. J Pharm Pract Res. (2017) 47:47–50. doi: 10.1002/jppr.1224

[28] Wolver SE Stultz JS Aggarwal A Thacker L Banas C. Provider and patient perceptions of an external medication history function. J Patient Saf. (2018) 14:234–40. doi: 10.1097/PTS.0000000000000197, PMID: 26101998

[29] Institute for Safe Medication Practices. Quick Safety Issue 26: Transitions of Care: Managing Medications (Updated April 2022). (2016). Available from: https://www.jointcommission.org/en-us/knowledge-library/newsletters/quick-safety/issue-26 (Accessed June 30, 2025).

[30] Canadian Patient Safety Institute ISMP Canada. Medication Reconciliation in Acute Care (2017). Available online at: https://www.ismp-Canada.org/download/MedRec/MedRec-AcuteCare-GSK-EN.pdf (Accessed June 30, 2025).

[31] Holt KM Thompson AN. Implementation of a medication reconciliation process in an internal medicine clinic at an academic medical center. Pharm (Basel). (2018) 6(2):26. doi: 10.3390/pharmacy6020026, PMID: 29587353 PMC6025090

[32] Llorca PM Abbar M Courtet P Guillaume S Lancrenon S Samalin L. Guidelines for the use and management of long-acting injectable antipsychotics in serious mental illness. BMC Psychiatry. (2013) 13:340. doi: 10.1186/1471-244X-13-340, PMID: 24359031 PMC3898013

[33] Farre A Heath G Shaw K Bem D Cummins C. How do stakeholders experience the adoption of electronic prescribing systems in hospitals? A systematic review and thematic synthesis of qualitative studies. BMJ Qual Saf. (2019) 28:1021–31. doi: 10.1136/bmjqs-2018-009082, PMID: 31358686 PMC6934241

[34] Keenan R Borycki EM Kushniruk AW. Computerized provider order entry and patient safety: A scoping review. Knowledge Manage E-Learning. (2021) 13:452–76. doi: 10.34105/j.kmel.2021.13.025

[35] Setareh S Rabiei R Mirzaei HR Roshanpoor A Shaabani M. Effects of guideline-based computerized provider order entry systems on the chemotherapy order process: A systematic review. Acta Inform Med. (2022) 30:61–8. doi: 10.5455/aim.2022.30.61-68, PMID: 35800912 PMC9226788

[36] Shamliyan TA Duval S Du J Kane RL. Just what the doctor ordered. Review of the evidence of the impact of computerized physician order entry system on medication errors. Health Serv Res. (2008) 43:32–53. doi: 10.1111/j.1475-6773.2007.00751.x, PMID: 18211517 PMC2323150

[37] Wien K Thern J Neubert A Matthiessen BL Borgwardt S. Reduced prevalence of drug-related problems in psychiatric inpatients after implementation of a pharmacist-supported computerized physician order entry system - a retrospective cohort study. Front Psychiatry. (2024) 15:1304844. doi: 10.3389/fpsyt.2024.1304844, PMID: 38654729 PMC11035719

[38] Carpenter J. Long-acting injectable antipsychotics: what to do about missed doses: use a stepwise approach based on the unique properties of the specific medication. Curr Psychiatry. (2018) 17:10–20.

[39] Emsley R Nuamah I Hough D Gopal S. Treatment response after relapse in a placebo-controlled maintenance trial in schizophrenia. Schizophr Res. (2012) 138:29–34. doi: 10.1016/j.schres.2012.02.030, PMID: 22446143

[40] Jaeger M Rossler W. Attitudes towards long-acting depot antipsychotics: A survey of patients, relatives and psychiatrists. Psychiatry Res. (2010) 175:58–62. doi: 10.1016/j.psychres.2008.11.003, PMID: 20004980

[41] Potkin S Bera R Zubek D Lau G. Patient and prescriber perspectives on long-acting injectable (Lai) antipsychotics and analysis of in-office discussion regarding lai treatment for schizophrenia. BMC Psychiatry. (2013) 13:261. doi: 10.1186/1471-244X-13-261, PMID: 24131801 PMC3819472

[42] Kane JM Schooler NR Marcy P Achtyes ED Correll CU Robinson DG. Patients with early-phase schizophrenia will accept treatment with sustained-release medication (Long-acting injectable antipsychotics): results from the recruitment phase of the prelapse trial. J Clin Psychiatry. (2019) 80(3):18m12456. doi: 10.4088/JCP.18m12546, PMID: 31050233

[43] Rubio JM Mychaskiw MA Lim S Suett M Wang Y Tian M. Predictors for initiation of atypical long-acting injectable antipsychotic agents in a commercial claims cohort of individuals with early-phase schizophrenia. J Clin Psychiatry. (2023) 84(2):22m14604. doi: 10.4088/JCP.22m14604, PMID: 36791360

[44] Weiden PJ Roma RS Velligan DI Alphs L DiChiara M Davidson B. The challenge of offering long-acting antipsychotic therapies: A preliminary discourse analysis of psychiatrist recommendations for injectable therapy to patients with schizophrenia. J Clin Psychiatry. (2015) 76:684–90. doi: 10.4088/JCP.13m08946, PMID: 25939027

[45] Fiorillo A Barlati S Bellomo A Corrivetti G Nicolo G Sampogna G. The role of shared decision-making in improving adherence to pharmacological treatments in patients with schizophrenia: A clinical review. Ann Gen Psychiatry. (2020) 19:43. doi: 10.1186/s12991-020-00293-4, PMID: 32774442 PMC7409631

[46] Polinski JM Kowal MK Gagnon M Brennan TA Shrank WH. Home infusion: safe, clinically effective, patient preferred, and cost saving. Healthc (Amst). (2017) 5:68–80. doi: 10.1016/j.hjdsi.2016.04.004, PMID: 28668202

[47] The National Association of Boards of Pharmacy (NABP). White and brown bagging emerging practices, emerging regulation. (2018). Available from: https://nabp.pharmacy/wp-content/uploads/2018/04/White-Bagging-and-Brown-Bagging-Report-2018_Final-1.pdf (Accessed June 30, 2025).

[48] Rumore MM. Pharmacy sees surge in legislative activity to regulate specialty drug dispensing practices in 2023. Pharm Pract Focus: Health Syst. (2023) 12(6):14–7. Available from: https://www.pharmacytimes.com/view/pharmacy-sees-surge-in-legislative-activity-to-regulate-specialty-drug-dispensing-practices-in-2023. (Accessed June 30, 2025)

[49] Soliman E Ranjan S Xu T Gee C Harker A Barrera A. A narrative review of the success of intramuscular gluteal injections and its impact in psychiatry. Biodes Manuf. (2018) 1:161–70. doi: 10.1007/s42242-018-0018-x, PMID: 30546922 PMC6267269

[50] Black RM Hughes TD Ma F Hudzik AA Shepherd G Ferreri S. Systematic review of community pharmacist administration of long-acting injectable antipsychotic medications. J Am Pharm Assoc (2003). (2023) 63:742–50 e3. doi: 10.1016/j.japh.2022.08.006, PMID: 36740528

[51] Boothby L Webb E Goodlett D Brown K Odinet J. Medication administration by inpatient pharmacists: innovative interdisciplinary care teams. Am J Health Syst Pharm. (2024) 82(11):e523–28. doi: 10.1093/ajhp/zxae374, PMID: 39656963

[52] Health Resources and Services Administration Bureau of Health Workforce. Behavioral Health Workforce, 2023. National Center for Health Workforce Analysis (2023). Available from: https://bhw.hrsa.gov/sites/default/files/bureau-health-workforce/state-of-the-behavioral-health-workforce-report-2024.pdf (Accessed June 30, 2025).

[53] Wang D Schneider-Thoma J Siafis S Qin M Wu H Zhu Y. Efficacy, acceptability and side-effects of oral versus long-acting- injectables antipsychotics: systematic review and network meta-analysis. Eur Neuropsychopharmacol. (2024) 83:11–8. doi: 10.1016/j.euroneuro.2024.03.003, PMID: 38490016

[54] Efthimiou O Taipale H Radua J Schneider-Thoma J Pinzon-Espinosa J Ortuno M. Efficacy and effectiveness of antipsychotics in schizophrenia: network meta-analyses combining evidence from randomised controlled trials and real-world data. Lancet Psychiatry. (2024) 11:102–11. doi: 10.1016/S2215-0366(23)00366-8, PMID: 38215784

[55] Drevin GR B. Ballot-Ragaru J Bonnot E Palayer M Gaconnet A Deguigne M. Death following an intramuscular injection of paliperidone: A case report. Toxicol Analytique Clin. (2020) 32:132–6. doi: 10.1016/j.toxac.2019.10.006

[56] Borojevic N Dawud M Xiao J Yun Y. Long-acting injectable paliperidone palmitate induced severe cutaneous allergic reaction in a patient with first episode delusional disorder tolerating oral paliperidone regimen: A case report. BMC Psychiatry. (2022) 22:734. doi: 10.1186/s12888-022-04347-7, PMID: 36434603 PMC9700998

[57] Perry R Wolberg J DiCrescento S. Anaphylactoid reaction to paliperidone palmitate extended-release injectable suspension in a patient tolerant of oral risperidone. Am J Health Syst Pharm. (2012) 69:40–3. doi: 10.2146/ajhp110230, PMID: 22180550

[58] Sampogna G Di Vincenzo M Giuliani L Menculini G Mancuso E Arsenio E. A systematic review on the effectiveness of antipsychotic drugs on the quality of life of patients with schizophrenia. Brain Sci. (2023) 13(11):1577. doi: 10.3390/brainsci13111577, PMID: 38002537 PMC10669728

[59] Perez-Revuelta JI Gonzalez-Saiz F Pascual-Pano JM Mongil-San Juan JM Rodriguez-Gomez C Munoz-ManChado LI. Shared decision making with schizophrenic patients: A randomized controlled clinical trial with booster sessions (Decide study). Patient Educ Couns. (2023) 110:107656. doi: 10.1016/j.pec.2023.107656, PMID: 36807126

[60] Wang GH Svensson M Shao H Vouri SM Park H. Cost-effectiveness analysis of monthly, 3-monthly, and 6-monthly long-acting injectable and oral paliperidone in adults with schizophrenia. J Manag Care Spec Pharm. (2023) 29:884–95. doi: 10.18553/jmcp.2023.29.8.884, PMID: 37523313 PMC10397333

[61] Schoretsanitis G Kane JM Correll CU Marder SR Citrome L Newcomer JW. Blood levels to optimize antipsychotic treatment in clinical practice: A joint consensus statement of the american society of clinical psychopharmacology and the therapeutic drug monitoring task force of the arbeitsgemeinschaft fur neuropsychopharmakologie und pharmakopsychiatrie. J Clin Psychiatry. (2020) 81(3):19cs13169. doi: 10.4088/JCP.19cs13169, PMID: 32433836

[62] Schoretsanitis G Baumann P Conca A Dietmaier O Giupponi G Grunder G. Therapeutic drug monitoring of long-acting injectable antipsychotic drugs. Ther Drug Monit. (2021) 43:79–102. doi: 10.1097/FTD.0000000000000830, PMID: 33196621

[63] Hiemke C Bergemann N Clement HW Conca A Deckert J Domschke K. Consensus guidelines for therapeutic drug monitoring in neuropsychopharmacology: update 2017. Pharmacopsychiatry. (2018) 51:9–62. doi: 10.1055/s-0043-116492, PMID: 28910830

[64] Fagiolini A Alfonsi E Amodeo G Cenci M Di Lella M Farinella F. Switching long acting antipsychotic medications to aripiprazole long acting once-a-month: expert consensus by a panel of italian and spanish psychiatrists. Expert Opin Drug Saf. (2016) 15:449–55. doi: 10.1517/14740338.2016.1155553, PMID: 26886162

[65] Guinart D Varma K Segal Y Talasazana N Correll CU Kane JM. Reasons for antipsychotic treatment switch: A systematic retrospective review of prescription records and prescriber notes. J Clin Psychiatry. (2022) 83(5):21m14272. doi: 10.4088/JCP.21m14272, PMID: 36070576

[66] Ehret MJ Carr CN Ehret MJ Carr CN. Discontinuing a long-acting injectable antipsychotic: what to consider. Curr Psychiatry. (2023) 22(2):35–8. doi: 10.12788/cp

[67] Pyle D Mendez-Reyes JE Esteban C Hazi A Andre M Yap S. Flowsheet template for the documentation of allergic reactions in infants and toddlers. J Allergy Clin Immunol Pract. (2024) 12:2221–2 e10. doi: 10.1016/j.jaip.2024.05.029, PMID: 39122335

[68] Belda-Rustarazo S Cantero-Hinojosa J Salmeron-Garcia A Gonzalez-Garcia L Cabeza-Barrera J Galvez J. Medication reconciliation at admission and discharge: an analysis of prevalence and associated risk factors. Int J Clin Pract. (2015) 69:1268–74. doi: 10.1111/ijcp.12701, PMID: 26202091

[69] Zhou H Ngune I Albrecht MA Della PR. Risk factors associated with 30-day unplanned hospital readmission for patients with mental illness. Int J Ment Health Nurs. (2023) 32:30–53. doi: 10.1111/inm.13042, PMID: 35976725

[70] Fonseca Barbosa J Gama Marques J. The revolving door phenomenon in severe psychiatric disorders: A systematic review. Int J Soc Psychiatry. (2023) 69:1075–89. doi: 10.1177/00207640221143282, PMID: 37209104 PMC10338701

[71] Cohen GR Friedman CP Ryan AM Richardson CR Adler-Milstein J. Variation in physicians’ Electronic health record documentation and potential patient harm from that variation. J Gen Intern Med. (2019) 34:2355–67. doi: 10.1007/s11606-019-05025-3, PMID: 31183688 PMC6848521

[72] Ebbers T Kool RB Smeele LE Dirven R den Besten CA Karssemakers LHE. The impact of structured and standardized documentation on documentation quality; a multicenter, retrospective study. J Med Syst. (2022) 46:46. doi: 10.1007/s10916-022-01837-9, PMID: 35618978 PMC9135789

[73] Clark NA Simmons J Etzenhouser A Pallotto EK. Improving outpatient provider communication for high-risk discharges from the hospitalist service. Hosp Pediatr. (2021) 11:1033–48. doi: 10.1542/hpeds.2020-005421, PMID: 34526327

[74] Schoretsanitis G Kane JM Correll CU Rubio JM. Predictors of lack of relapse after random discontinuation of oral and long-acting injectable antipsychotics in clinically stabilized patients with schizophrenia: A re-analysis of individual participant data. Schizophr Bull. (2022) 48:296–306. doi: 10.1093/schbul/sbab091, PMID: 34355232 PMC8886604

[75] Koparal B Unler M Utku HC Candansayar S. Revolving door phenomenon and related factors in schizophrenia, bipolar affective disorder and other psychotic disorders. Psychiatr Danub. (2021) 33:18–26. doi: 10.24869/psyd.2021.18, PMID: 33857036

[76] Smith TE Haselden M Corbeil T Wall MM Tang F Essock SM. Effect of scheduling a post-discharge outpatient mental health appointment on the likelihood of successful transition from hospital to community-based care. J Clin Psychiatry. (2020) 81(5):20m13344. doi: 10.4088/JCP.20m13344, PMID: 32936543 PMC10476606

[77] Khaira M Mathers A Benny Gerard N Dolovich L. The evolving role and impact of integrating pharmacists into primary care teams: experience from ontario, Canada. Pharm (Basel). (2020) 8(4):234. doi: 10.3390/pharmacy8040234, PMID: 33297509 PMC7768418

[78] Cai C Kozma C Patel C Benson C Yunusa I Zhao P. Adherence, health care utilization, and costs between long-acting injectable and oral antipsychotic medications in south carolina medicaid beneficiaries with schizophrenia. J Manag Care Spec Pharm. (2024) 30:549–59. doi: 10.18553/jmcp.2024.30.6.549, PMID: 38824623 PMC11144998

[79] Doshi JA Li P Geng Z Seo S Patel C Benson C. Out-of-pocket costs for long-acting injectable and oral antipsychotics among medicare patients with schizophrenia. Psychiatr Serv. (2024) 75:333–41. doi: 10.1176/appi.ps.20230142, PMID: 37960866

[80] Tyler N Wright N Waring J. Interventions to improve discharge from acute adult mental health inpatient care to the community: systematic review and narrative synthesis. BMC Health Serv Res. (2019) 19:883. doi: 10.1186/s12913-019-4658-0, PMID: 31760955 PMC6876082

[81] Sajatovic M Ross R Legacy SN Byerly M Kane JM DiBiasi F. Initiating/maintaining long-acting injectable antipsychotics in schizophrenia/schizoaffective or bipolar disorder - expert consensus survey part 2. Neuropsychiatr Dis Treat. (2018) 14:1475–92. doi: 10.2147/NDT.S167485, PMID: 29922063 PMC5997122

[82] Holmgren AJ Esdar M Husers J Coutinho-Almeida J. Health information exchange: understanding the policy landscape and future of data interoperability. Yearb Med Inform. (2023) 32:184–94. doi: 10.1055/s-0043-1768719, PMID: 37414031 PMC10751121

[83] Li E Clarke J Ashrafian H Darzi A Neves AL. The impact of electronic health record interoperability on safety and quality of care in high-income countries: systematic review. J Med Internet Res. (2022) 24:e38144. doi: 10.2196/38144, PMID: 36107486 PMC9523524

[84] Colaiaco B Roth CP Ganz DA Hanson M Smith P Wenger NS. Continuity of information between mental health and primary care providers after a mental health consultation. Psychiatr Serv. (2018) 69:1081–6. doi: 10.1176/appi.ps.201800025, PMID: 30041587

[85] Munck LK Hansen KR Molbak AG Balle H Kongsgren S. The use of shared medication record as part of medication reconciliation at hospital admission is feasible. Dan Med J. (2014) 61:A4817., PMID: 24814735

