# Supplementary material for: Optimizing long-acting injectable antipsychotic safety and care continuity through documentation best practices
Source: Front Psychiatry. 2025 Sep 1;16:1659290. doi: 10.3389/fpsyt.2025.1659290 (PMC12434062; doi:10.3389/fpsyt.2025.1659290)
Supplement: Supplementary file 1 [file SupplementaryFile1.pdf]

Supplementary Material

The author(s) verify and take full responsibility for the use of generative AI in the preparation of this manuscript. OpenAI, GPT-4o (Omni) was used to formulate the abstract and create an initial draft for the LAI-AP templates provided in the supplementary materials before being adjusted by the authors to cover additional pertinent information.

Prompts included "Create a structured abstract for this manuscript," "Can you please create a template with the following checklist? The duration of corresponding oral medication was sufficient to determine safety and efficacy prior to starting the LAI-AP; The chosen dose of the LAI-AP corresponds to the oral antipsychotic dose on which the patient was stabilized; The correct loading or re-initiation dose sequence has been ordered for the patient (if applicable); The corresponding oral antipsychotic medication has been discontinued or has been continued for the proper overlap duration after LAI-AP initiation The chosen dose of the LAI-AP corresponds to the patient's renal function (if applicable); The date and dose of the last LAI-AP has been noted in the chart (if applicable); The patient has access to the LAI-AP on an outpatient basis (affordability and accessibility); The outpatient prescription for the LAI-AP", and "Can you also provide a template for the following: Review the administration instructions within the package insert or on the manufacturer's LAI-AP box packaging; Ensure the proper steps are completed: The administration site has been identified and cleaned for injection, The needle size and length are correct for the patient's body habitus, The injection technique has been correctly identified (ex: intramuscularly, Z-track method), Ensure barcode medication administration occurs every time; Document the following: Dose administered, Administration site, Needle size/length utilized, Name of clinician providing injection, Date/time of administration, Patient refusal of the injection (if applicable)."
